# Motor cortical functional connectivity changes due to short-term immobilization of upper limb: an fNIRS case report

**DOI:** 10.3389/fresc.2023.1156940

**Published:** 2023-05-17

**Authors:** Arun Karumattu Manattu, Jordan A. Borrell, Christopher Copeland, Kaitlin Fraser, Jorge M. Zuniga

**Affiliations:** ^1^ Department of Biomechanics, University of Nebraska at Omaha, Omaha, NE, United States; ^2^ Center for Biomedical Rehabilitation and Manufacturing, University of Nebraska at Omaha, Omaha, NE, United States

**Keywords:** fNIRS (functional near infrared spectroscopy), resting state connectivity, immobilization, plasticity, functional reorganization

## Abstract

**Introduction:**

A short-term immobilization of one hand affects musculoskeletal functions, and the associated brain network adapts to the alterations happening to the body due to injuries. It was hypothesized that the injury-associated temporary disuse of the upper limb would alter the functional interactions of the motor cortical processes and will produce long-term changes throughout the immobilization and post-immobilization period.

**Methods:**

The case participant (male, 12 years old, right arm immobilized for clavicle fracture) was scanned using optical imaging technology of fNIRS over immobilization and post-immobilization. Pre-task data was collected for 3 min for RSFC analysis, processed, and analyzed using the Brain AnalyzIR toolbox. Connectivity was measured using Pearson correlation coefficients (*R*) from NIRS Toolbox's connectivity module.

**Results:**

The non-affected hand task presented an increased ipsilateral response during the immobilization period, which then decreased over the follow-up visits. The right-hand task showed a bilateral activation pattern following immobilization, but the contralateral activation pattern was restored during the 1-year follow-up visit. Significant differences in the average connection strength over the study period were observed. The average Connection strength decreased from the third week of immobilization and continued to be lower than the baseline value. Global network efficiency decreased in weeks two and three, while the network settled into a higher efficient state during the follow-up periods after post-immobilization.

**Discussion:**

Short-term immobilization of the upper limb is shown to have cortical changes in terms of activations of brain regions as well as connectivity. The short-term dis-use of the upper limb has shifted the unilateral activation pattern to the bilateral coactivation of the motor cortex from both hemispheres. Resting-state data reveals a disruption in the motor cortical network during the immobilization phase, and the network is reorganized into an efficient network over 1 year after the injury. Understanding such cortical reorganization could be informative for studying the recovery from neurological disorders affecting motor control in the future.

## Introduction

The human brain adapts to the modifications happening to the body by reorganizing the connections between neurons. This process of brain plasticity initially involves a short-term rearrangement of existing functional connections (1). Over repeated exposure to such modifications, the structural changes may contribute to the change in connectivity within the brain (2). The rearrangements of the neuronal connections that characterize brain plasticity are generally understood to affect the individual's well-being (3) positively. In the case of motor recovery from brain injuries like stroke due to long-term rehabilitation therapies, motor and cognitive skill learning during brain development reflect adaptive plasticity (4, 5). In contrast, if the connectivity changes are detrimental to the organic well-being of the individual, plasticity could be unsound (6). For example, studies have reported that detrimental neuroplastic changes inducing neuropathic pain and task-induced dystonia are attributed to maladaptive plastic brain changes (7, 8). Functional near-infrared spectroscopy (fNIRS) is identified as one of the brain imaging modalities that can monitor the functional activations of neural networks, as well as many other neuroplastic cortical changes in the brain during a task and resting conditions (9–11). Apart from the brain activation during a particular task in response to a stimulus, it is also possible to extract neural signals during resting conditions revealing intrinsic neural network arrangements (12). The motor cortex has been widely studied using fNIRS since this modality offers portable measurement of brain signals for movement-related studies (13). Researchers have used fNIRS to extend the boundaries of motor behavior research to examine cortical activity and determine spatial characteristics of activity during motor tasks (14).

A period of immobilization significantly affects musculoskeletal motor functions and causes significant changes in the brain (15). Sensorimotor skills and events are known to play an important role in shaping the cortical motor representation (16). These representations are typically flexible or dynamic in response to the amount of use or disuse of the corresponding body parts (17). Huber et al. observed that there is a substantial decrease in neuronal activity revealed by peripheral nerve stimulation evoked potentials in the hand areas of the right hemisphere due to immobilization of the arm for 12 consecutive hours (18). Another study revealed that the motor performance on the reach-to-grasp task was regained to baseline level after a few trials of practice post the immobilization of the arm for 10 h (19). Motor-evoked potential studies also explored the interhemispheric interactions of motor cortices during immobilized upper limb conditions. A decreased interhemispheric inhibition and motor excitability in the left hemisphere were observed when the right upper arm and hand were immobilized (19). Studies that reported the structural changes caused by the immobilization of the right upper limb observed that there was a reduction in the cortical thickness in the left sensorimotor cortex and over the left corticospinal tract after 16 days of immobilization while the ipsilateral motor cortical thickness was increased during the phase of immobilization (20). Non-human primate studies and recent studies on human cohorts also inform us that reversible cortical changes are possible with very short-term immobilization, and can selectively alter neural plasticity (21, 22). Thus, there is a critical gap describing the motor cortex changes before and after immobilization during resting and task conditions.

The current study aims to investigate the motor cortical functional connectivity for the immobilized upper right extremity due to a right clavicle fracture. The resting state data were collected to study the intrinsic motor cortex connectivity and the alterations to those connections during the immobilization and recovery phases. It was hypothesized that the injury-associated temporary disuse of the upper limb would alter the functional interactions of the motor cortical processes and will produce changes in the neural networks before and after the immobilization period. Our hypothesis was based on previous investigations that have reported the functional and structural brain reorganization in the sensorimotor cortex (2, 19, 20).

## Methodology

### Case description

**Figure 1:**
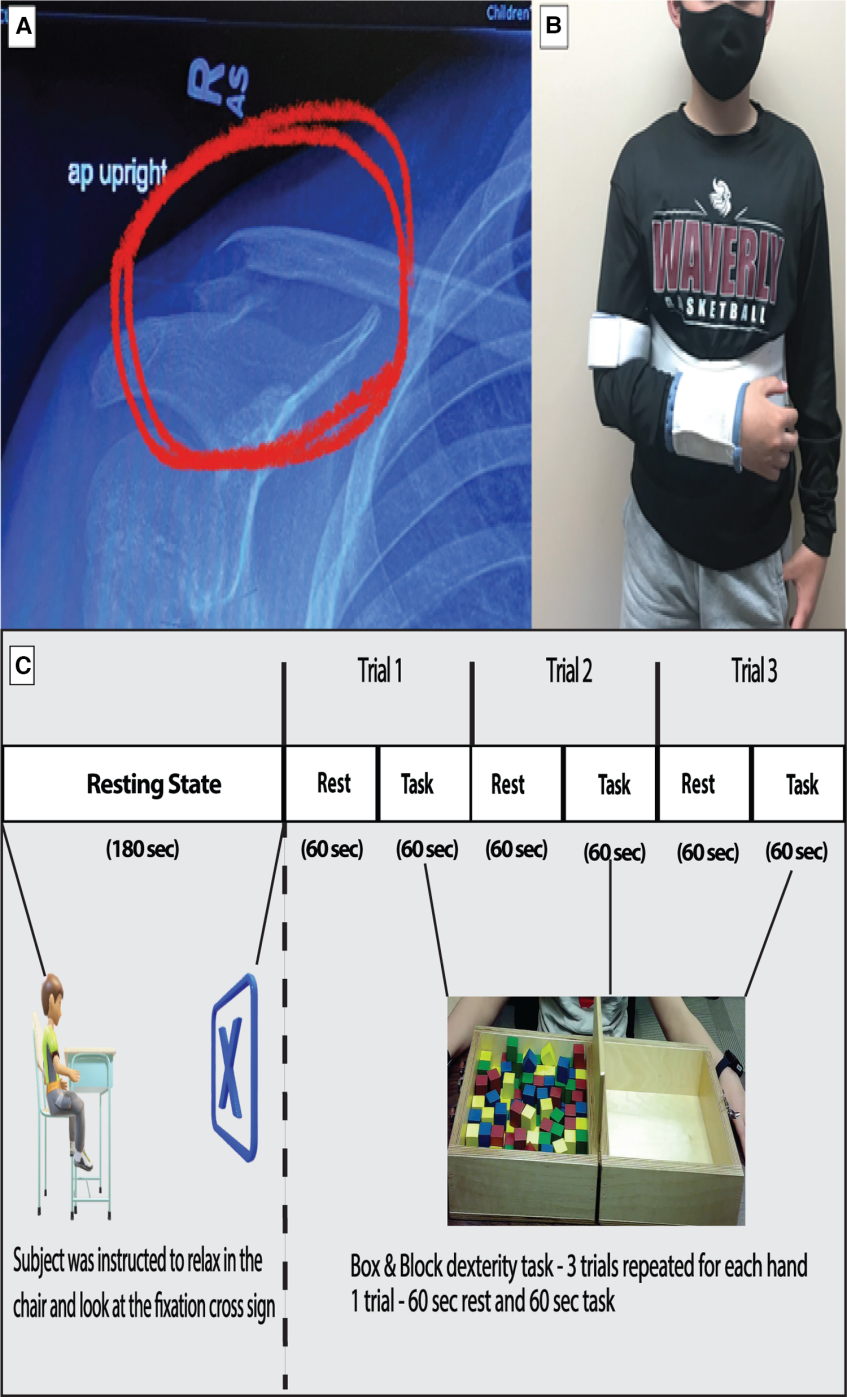
(**A**) Shows the fractured right clavicle of the subject. (**B**) Show the subject with right hand immobilized with a sling. (**C**) The experimental design of the study is depicted with duration of resting period for connectivity analysis and the task period for box and block task used for task-based analysis.

The case participant was a 12-year-old male (Height: 156.94 cm and Weight: 49.9 kg) diagnosed with a lateral right clavicle fracture and prescribed an arm sling immobilizer (Figure 1A and 1B). The participant was enrolled in a control group for a different brain imaging study. The participant's entire dominant right arm was immobilized 4 days after his initial laboratory visit due to the injury. He was taken to the emergency room the same day, where his arm was immobilized. On Monday (3 days after immobilization), the participant visited our laboratory, where another brain imaging acquisition was performed. Immobilization of the right arm continued for 3 weeks. Due to the injury, the participant had to become dependent on the non-affected, non-dominant left arm for the performance of activities of daily living during the 3-week immobilization period. The participants had no apparent or diagnosed traumatic brain injury or neurological issues while undergoing the study. The study was approved by the University of Nebraska at Omaha Review Board.

### Data acquisition

Data were collected using a continuous wave 24-channel fNIRS system (NIRSport2, NIRx Medical Technologies, LLC, Berlin, Germany). A dual-wavelength (760 and 850 nm) LED source shines light on the subject's scalp. Data were sampled at 10.125 Hz. The cap held eight sources and eight detectors (∼3 cm distance from the source) arranged in a specific montage covering the motor cortex of both hemispheres. The cap was positioned on the head following the 10–20 international system. The center of the cap was aligned with the vertex (Cz), and lateral channels covered the area around the C3 and C4 landmarks which have been shown to detect motor activity that drives hand and arm movement (14). The eight sources and eight detectors were arranged to form twenty channels to measure the hemodynamic response in both hemispheres of the primary motor cortex (M1), supplementary motor area (SMA), and premotor cortex (PMC) were recorded (23).

The data collection was a longitudinal process. The fNIRS data were collected in six different sessions: Baseline, Week 1–Week 3, Sixth-month post immobilization, and 1-year post immobilization. During the baseline visit, the participant underwent brain imaging using fNIRS. This included a three-minute resting-state period and task period, as shown in the protocol (Figure 1C). A relaxed state helps to stabilize the functional connectivity measures obtained from the data (24). This was followed by a box and block (B&B) task performed to assess gross manual dexterity (25). The subject moved one block at a time from one side over a partition and dropped the blocks on the adjacent side for three trials of sixty seconds each. During the immobilization visits, the task session was performed by the unaffected hand alone.

### Data processing

#### Block averaging analysis

**Figure 2:**
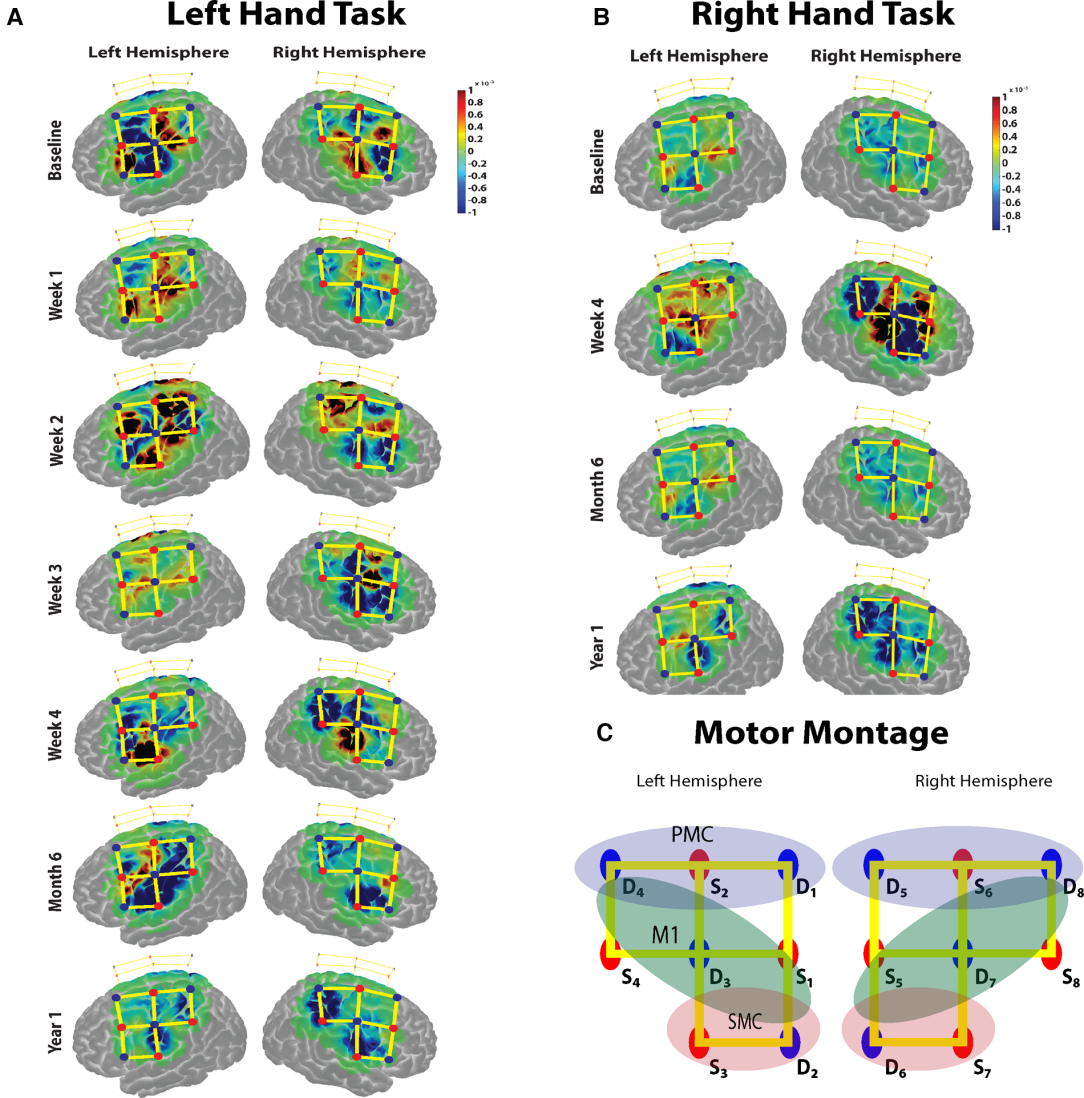
(**A,B**) The task based cortical activation (change in oxyhemoglobin) for the non-affected (left) hand and the immobilized (right) hand for each session is shown. (**C**) The montage used for the data acquisition over the motor cortex. Red dots represent the sources, blue dots represent the detectors, and the yellow lines represent the channels of measurement. The regions of interest for which the task activation was calculated are marked in colored oval shapes. Blue shaded oval shape- pre-motor cortex (PMC); Green shaded oval shape- primary motor cortex (M1); Red shaded oval shape- somatosensory cortex (SMC) channels.

Data were analyzed using the open-sourced Homer3 (v1.26) Toolbox (26). Hemodynamic data were reconstructed on atlas anatomy utilizing the AtlasViewer (v2.12.4) Toolbox (27). The raw fNIRS signals were first converted into changes in optical density by taking the logarithm of the signal. A PCA filter and a bandpass filter (passband: 0.01–0.20 Hz) were applied to the optical density data to remove motion artifacts and physiological noise. The concentrations of oxygenated hemoglobin (HbO) and deoxygenated hemoglobin (HbR) were then obtained using the modified Beer-Lambert law. A block-averaging approach to each task/stimulus event then estimated the hemodynamic response function (HRF). The processed oxygenated hemodynamic (HbO) data were exported from Homer3 and analyzed separately. Regions of interest were determined by grouping each channel based on the MNI coordinates determined in AtlasViewer. The beta values from a time range of (5–60) sec was extracted from each channel and averaged for each region of interest (ROI) (Figure 2C).

#### Resting-state analysis

Three minutes of pre-task data were collected for resting-state functional connectivity (RSFC) analysis. The raw data were then pre-processed and analyzed with the help of the NIRS Brain AnalyzIR toolboxes (28). A PCA filter was employed to remove the motion correction. The data were down-sampled to 1 Hz, optical density was estimated, and the Beer-Lambert Law was used to calculate oxygenated hemoglobin data. The Pearson correlation coefficients (R) were calculated using the NIRS Toolbox's “connectivity” module, which employs an autoregressive correlation function to help reduce the confounding effects of physiological phenomena that can lead to false positive results (29). The correlation values were converted to *Z* values using Fisher's transformation (30), which normalized the Pearson correlation coefficients' variance.

#### Network construction

A network is a collection of nodes and edges, whereas in a macroscale brain network, nodes indicate the channel locations used in the montage, and the edges are the correlation of the time series between each channel. The adjacency matrices were calculated for the entire range of sparsity threshold (0–1.0 with an interval of 0.1) and binarized at each threshold for calculating the following network properties.

(i) Connectivity strength: It is the sum of the connectivity weights of the edges attached to each node. The mean value of the connectivity strength across all the channels will be a metric that can be compared across the immobilization phase to understand the overall changes in the intrinsic connections.
(ii) Network efficiency: For a brain network (*G*), the global efficiency is defined as $E_{g}(G)=\frac{1}{N(N-1)}\underset{i\neq j\in G\;}{\sum }\frac{1}{L_{ij}}$ where *N* is the number of nodes and *L_ij_* is the shortest path length between *i*th and *j*th nodes in the network *G*. E_g_ is the efficiency of information exchange in a parallel system in which all nodes are capable of concurrently exchanging information via shortest paths (31).
(iii) Nodal degree: It quantifies the total number of edges incident to a node in a binary network. A smaller number of essential nodes receive the majority of connectivity, designating them as nodal hubs that encourage network integration (32).

#### Statistical analysis

For the analysis of task data, a student's *t*-test was used to compare the means of HbO values from the ROIs to obtain *p*-values. A Bonferroni correction was used to control multiple comparisons to get *q*-values. HbR data were not analyzed or displayed for this study. For the resting data, a one-way repeated measure of ANOVA was conducted to investigate the overall differences in the connectivity strength values for the different data collection time points in the whole study. *Post hoc* two-sample *t*-tests were performed to test the differences between specific data collection time points. A corrected *p*-value of 0.05 was considered significant.

## Results

### Gross manual dexterity task

The case participant moved more blocks with the non-dominant (intact hand) during immobilized sessions and used both hands during the follow-up visits, conducting the task at his own determined pace. There was a steady increase in the number of blocks moved (Table 1).

**Table 1:** Number of blocks moved during the box and block task.

		Baseline	Week1	Week2	Week3	Week4	6 months	12 months
Left hand	Trial 1	64	63	71	64	68	68	77
	Trial 2	67	69	69	64	73	77	79
	Trial 3	64	68	71	72	84	74	81
	Mean	65	66.66	70.33	66.66	75	73	79
Right hand (Immobilized)	Trial 1	58				72	74	75
	Trial 2	66				72	74	73
	Trial 3	68				73	77	76
	Mean	64				72.33	74	74.66

### Motor cortical response for B&B task

The baseline motor cortical response results show a higher change in HbO in the left M1 area for the right-hand task. For the left-hand task, there was an increase in change in HbO in contralateral M1 areas and ipsilateral S1 regions (Figure 2A). During the immobilization period for weeks 1, 2 and 3, there was a significant increase in the average ipsilateral motor cortical response (Bonferroni corrected *p* < 0.05) compared to the baseline response for the non-affected hand task. The week 4 task response showed a reduced ipsilateral response for the left-hand task. The bilateral response of the left-hand task was retained after the immobilization period during the 6-month follow-up measurement but was significantly reduced during the second follow-up at 12 months. For the immobilized hand, after immobilization (week 4), strong bilateral activation was observed. However, the 6-month follow-up measurement showed a reduction in bilateral activation for the right-hand task and persisting with contralateral activation pattern even at the 12-month follow-up (Figure 2B). Thus, the non-affected hand task produced a baseline contralateral cortical response during the 6-month follow-up visit after a 3-week immobilization of the right hand. The immobilized right hand maintained the contralateral cortical response during the 6- and 12-month follow-up visits.

### Resting-state functional connectivity

#### Connectivity strength

**Figure 3:**
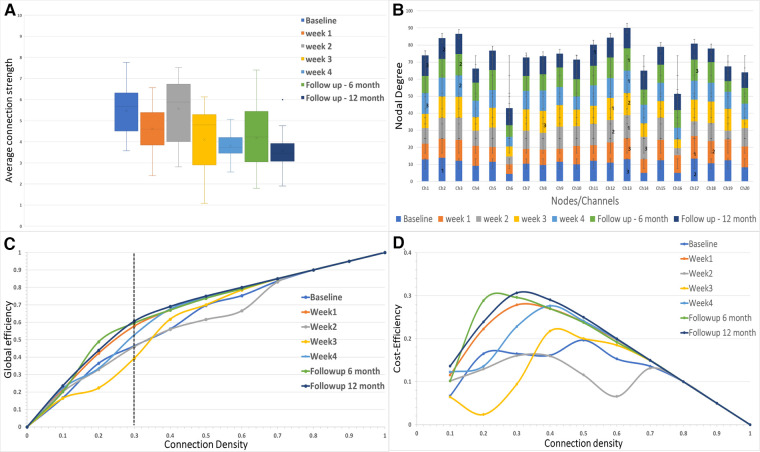
(**A**) The average connectivity strength between the channels is shown for each of the sessions in the study. (**B**) The nodal degree for each channel is shown in the stacked bar chart where each stack represents one session for each channel. The numbers 1, 2 and 3 represent the largest hubs in the network for each session. (**C,D**) The global network efficiency and cost efficiency as function of connection density (cost).

The mean functional connectivity strength was calculated each week for all the channels. It was observed that the average connection strength was significantly different [*F* (6,133) = (7.953), *p* < 0.005] for all the weeks of measurement (Figure 3A). The *post hoc* tests revealed that connection strength significantly decreased when compared to baseline for week 3 [*t* (38) = 3.087, Bonferroni corrected *p* < 0.05], week 4 [*t* (38) = 5.495, Bonferroni corrected *p* < 0.05], sixth-month follow-up [*t* (38) = 3.018, Bonferroni corrected *p* < 0.05] and 12-month follow-up [*t* (38) = 5.643, Bonferroni corrected *p* < 0.05]. There was no significant difference in connection strength between the final week and the follow-up months after immobilization.

#### Network efficiency

The network efficiency of the motor cortex is shown in Figure 3C for the entire range of connection densities (0–1.0). At 30% connection density, global efficiency at the 12-month follow-up showed a maximum increase (∼14%) from the baseline. For the immobilization period, Week 1 showed ∼11% difference (Figure 3C). Week 3 showed a decrease (∼6%) in network efficiency compared to the baseline. Cost-Efficiency as a function of connection density is shown in Figure 3D.

#### Nodal degree of the network

The degree distribution of each node is shown in Figure 3B. In the baseline measurement, Channel 2 had the maximum mean degree (largest hub) over the range of connection density thresholds (mean ± SD = 13.9 ± 4.09). The largest hub shifted to the right hemisphere during the immobilization period and remained in the right hemisphere for the follow-up measurements (Channel 17-Week 1; Channel 13-Week 2; Channel 12-Week 3; Channel 13-Week 4; Channel 13-followup 6 months; Channel 13-followup 12 months).

## Discussion

The current case report examines the changes in cortical responses to a task and resting conditions before, during, and after right upper limb immobilization. The primary findings of the study are: (i) The non-affected left-hand task presented an increased ipsilateral response during the immobilization period, which then decreased over the follow-up visits. The right-hand task showed a bilateral activation pattern immediately after the immobilization period, but the contralateral activation pattern was restored during the 1-year follow-up visit. (ii) During the resting period, the overall average strength of connectivity, network efficiency, and nodal degree in the motor cortex decreased during the immobilization period as opposed to the baseline measurement. The case study investigates the reformation of functional motor area network using resting state fNIRS unlike the previous works.

The task based baseline motor cortex measurement of the participant yielded a typical contralateral response for the gross dexterity movement of both hands, as reported in previous investigations (25, 33). However, the temporary disuse of the right hand due to the injury disturbed the contralateral cortical response resulting in a predominantly bilateral response just after the immobilization. This was reversed to a contralateral pattern after 1 year of immobilization. These findings are consistent with previous investigations that have shown that a period of limb immobilization causes significant neuroplastic changes in response to the amount of use or disuse of the corresponding body parts (14, 16). The decrease in neuronal activity found in the contralateral hemisphere during short-term immobilization regimes has been explained by the increase in bilateral control resulting in a more diffused cortical response in the former dominant contralateral hemisphere (16–19). This is supported by reports of decreased interhemispheric inhibition, which facilitates and promotes bilateral control, and reduced motor excitability in the left hemisphere when the right upper arm was immobilized (34). A reduction in the contralateral cortical thickness in the left sensorimotor cortex and left corticospinal tract after long-term arm immobilization, along with increases in the ipsilateral cortical thickness during the phase of immobilization was reported earlier further support this hypothesis and our findings during task performance (20).

The resting brain connectivity analysis was evaluated during the resting period before the task performance to capture endogenous and spontaneous network activity. To accomplish this objective, we used brain connectivity outcomes, including connection strength, the efficiency of the network, and nodal degree. The average connectivity strength lays out an overall change in the interhemispheric connectivity of the motor cortex (35). The immobilization of the right upper limb resulted in an increased interhemispheric connection during week two compared to the baseline measurement. This shows higher interhemispheric interaction as the ipsilateral regions tend to compensate for the disuse of the hand due to immobilization. Once the immobilization was eased out, the interhemispheric connectivity dropped and remained lower than baseline during follow-ups indicating contralateral regions taking back the control of the hand. These findings are consistent with the rapid and persistent reorganization of the brain expressed in white matter changes induced by immobilization reported by Langer et al. (20).

The intrinsic network efficiency decreased during the immobilization weeks at lower connection densities. The cost efficiency (∼30% connection density) for the relatively sparse network configuration is a point after which an increase in edges will not yield a proportional increase in efficiency. Increased functional network efficiency reported towards the 6- and 12-month follow-up sessions at this lower cost (∼20% to 30% connections) implies that the wiring volume and metabolic resources have been reduced compared to the immobilization period (35, 36, 37). The evaluation of nodal degree indicates the presence of hubs (channels with maximum connections), suggesting an increase in network interaction. The shift in the most prominent hub during immobilization could be due to the use of a non-dominant hand.

The case study's findings indicate that immobilizing the dominant hand alters the motor cortical response for the immobilized hand, which may be attributed to compensatory use of the non-dominant (unaffected) arm during the immobilization phase. Changes in intrinsic connectivity measures within the motor cortex reflect the disuse of the dominant hand and the adaptive use of the non-dominant left hand. Though the case report is limited in sample size for validating the findings, this understanding of changes in cortical mechanisms during short-term immobilization may be fostered by larger sample sized future studies and aid in developing rehabilitation strategies for recovering from motor control disorders.

## Data Availability

The raw data supporting the conclusions of this article will be made available by the authors, without undue reservation.
